# Electroencephalographic spectral power as a marker of cortical function and disease severity in girls with Rett syndrome

**DOI:** 10.1186/s11689-019-9275-z

**Published:** 2019-07-31

**Authors:** Katherine J. Roche, Jocelyn J. LeBlanc, April R. Levin, Heather M. O’Leary, Lauren M. Baczewski, Charles A. Nelson

**Affiliations:** 1 Laboratories of Cognitive Neuroscience, Division of Developmental Medicine, Boston Children’s Hospital, Harvard Medical School, 1 Autumn Street, Boston, MA 02215 USA; 2 Harvard-MIT Division of Health Sciences and Technology, Cambridge, MA USA; 3 F.M. Kirby Neurobiology Center, Neurology Department, Harvard Medical School, Boston, MA USA; 4 Department of Neurology, Boston Children’s Hospital, Boston, MA USA; 5 Graduate School of Education, Harvard University, Cambridge, MA USA

**Keywords:** Rett syndrome, EEG, Electroencephalography, Spectral power, Electrophysiology, Biomarker

## Abstract

**Background:**

Rett syndrome is a neurodevelopmental disorder caused by a mutation in the X-linked *MECP2* gene. Individuals with Rett syndrome typically develop normally until around 18 months of age before undergoing a developmental regression, and the disorder can lead to cognitive, motor, sensory, and autonomic dysfunction. Understanding the mechanism of developmental regression represents a unique challenge when viewed through a neuroscience lens. Are circuits that were previously established erased, and are new ones built to supplant old ones? One way to examine circuit-level changes is with the use of electroencephalography (EEG). Previous studies of the EEG in individuals with Rett syndrome have focused on morphological characteristics, but few have explored spectral power, including power as an index of brain function or disease severity. This study sought to determine if EEG power differs in girls with Rett syndrome and typically developing girls and among girls with Rett syndrome based on various clinical characteristics in order to better understand neural connectivity and cortical organization in individuals with this disorder.

**Methods:**

Resting state EEG data were acquired from girls with Rett syndrome (*n* = 57) and typically developing children without Rett syndrome (*n* = 37). Clinical data were also collected for girls with Rett syndrome. EEG power across several brain regions in numerous frequency bands was then compared between girls with Rett syndrome and typically developing children and power in girls with Rett syndrome was compared based on these clinical measures. 1/*ƒ* slope was also compared between groups.

**Results:**

Girls with Rett syndrome demonstrate significantly lower power in the middle frequency bands across multiple brain regions. Additionally, girls with Rett syndrome that are postregression demonstrate significantly higher power in the lower frequency delta and theta bands and a significantly more negative slope of the power spectrum. Increased power in these bands, as well as a more negative 1/ƒ slope, trended with lower cognitive assessment scores.

**Conclusions:**

Increased power in lower frequency bands is consistent with previous studies demonstrating a “slowing” of the background EEG in Rett syndrome. This increase, particularly in the delta band, could represent abnormal cortical inhibition due to dysfunctional GABAergic signaling and could potentially be used as a marker of severity due to associations with more severe Rett syndrome phenotypes.

## Background

Rett syndrome (RTT) is a rare, progressive neurodevelopmental disorder occurring almost exclusively in females that is caused by a mutation in the X-linked methyl-CpG binding protein 2 (*MECP2*) gene [1]. The disorder is generally marked by a period of seemingly typical development until 6 to 18 months of age followed by a developmental regression and the emergence of features such as stereotypies, motor dysfunction, loss of expressive language, intellectual disability, and epilepsy [2–4]. The objective assessment of brain function in individuals with RTT, including cognition and sensory processing, can be difficult due to problems with purposeful hand use and verbal communication, and there remains a need for objective biomarkers to assess cortical function and disease severity in this population.

*MECP2* codes for a transcriptional regulator required for normal postnatal gene expression in the brain as well as the formation of neuronal circuits [5]. Studies of mouse models of Rett syndrome have demonstrated that loss of functional MECP2 can lead to impaired neuronal maturation, altered GABAergic signaling, and ultimately an inability to maintain a normal excitatory and inhibitory balance within the brain [5–8]. Dysfunctional neuronal communication and a resulting cortical hyperexcitability are thought to contribute to the development of many of the symptoms seen in mouse models of the disorder as well as girls with RTT [7, 9, 10].

Computed tomography (CT), magnetic resonance imaging (MRI), and electroencephalography (EEG) have all emerged as tools to assist in the diagnosis and assessment of neurological disorders; however, EEG is perhaps the most scalable and affordable of these tools [11]. Using EEG, it is possible to study neural oscillations, or the rhythmic fluctuations in excitability of large populations of neurons. The generation of these neural oscillations is thought to be dependent on the excitatory and inhibitory balance of the cortex, and these oscillations have been linked to many processes in the brain, including sensory perception, memory, and cognition [12]. Various characteristics of the background EEG and power spectrum have been shown to be abnormal in numerous neurological and psychiatric disorders, and EEG abnormalities have been described in both mouse models of Rett syndrome and in girls with the disorder [13–16] However, for a number of conditions, including Rett syndrome, the power spectral characteristics as well as the association between disease severity and EEG findings, particularly power spectral data, are unknown.

In this study, we aimed to characterize the differences in baseline (resting) EEG spectral power as well as the slope of the power spectrum between girls with Rett syndrome and typically developing children. As there is currently a need for an unbiased, noninvasive biomarker to assess cortical function as well as disease severity and progression in girls with RTT, our secondary and tertiary aims were exploratory analyses designed to characterize the stability of EEG power over time in girls with RTT and to relate both spectral power and the slope of the power spectrum to data on both disease severity and cognitive function in girls with RTT in order to examine the association between power and various features of the disease in this population and to better understand the neural mechanisms underpinning this disorder.

## Methods

### Participants

Participants were recruited from ongoing Rett syndrome studies in the Boston Children’s Hospital Laboratories of Cognitive Neuroscience and Department of Neurology. Data were also acquired from several RTT participants in phase one (*n* = 8 RTT) and phase two (*n* = 24 RTT) of the Boston Children’s Hospital Insulin-like growth factor 1 clinical trial during the initial study visit prior to treatment with the study drug or placebo [17].

Baseline EEG data were successfully collected and analyzed from 57 girls with Rett syndrome and group of 37 female typically developing (TD) children. Data from several participants were excluded from EEG processing due to refusal of EEG lead placement or pulling at the leads during recording (*n* = 3 RTT and 1 TD), seizure activity during the recording (*n* = 1 RTT), and male gender (*n* = 1 RTT). Data from several participants were excluded during processing due to a lack of sufficient usable 1-s segments (< 30) as a result of excessive artifact (*n* = 13 RTT and 2 TD). There was no significant difference in age between the RTT (median = 51, interquartile range = 31, range = 23–131 months) and TD (median = 44, interquartile range = 29, range = 23–123 months) groups.

For several participants (*n* = 11 RTT and 18 TD), EEG data were also collected at a second study visit several months after the first. Data from the second study visit was only utilized in analyses of differences in EEG power over time, not in analyses of overall group differences in EEG power. There was no significant difference in length of time between visits in the RTT (median = 12, interquartile range = 4, range = 11–19 months) and TD (median = 15, interquartile range = 5, range = 9–18 months) groups.

### Clinical/behavioral assessments

To assess the impact of various phenotypic features of Rett syndrome on baseline EEG characteristics, several girls with RTT (*n* = 49) were grouped based on disorder stage. Girls were placed into two groups based on timing of most recent skill loss in the gross motor, fine motor, expressive language, receptive language, or adaptive skill domains. Girls with a skill loss in any of these areas in the 12 months prior to data acquisition were classified as being in active regression (AR, *n* = 20), and girls with no skill losses within 12 months were categorized as being postregression (PR, *n* = 29). This assessment was based on thorough developmental and medical history questionnaires completed by primary caregivers as well as a physical examination by a pediatric neurologist.

A majority of RTT participants (*n* = 47) were assigned a clinical severity score (CSS) based on medical history and assessment by a physician (median = 20, interquartile range = 8, range = 13–32). The CSS includes 13 measures specific to the RTT phenotype and assesses both current symptom severity as well as disease course [18, 19]. In this study, only composite (total) scores were considered. Some participants with RTT (*n* = 47) were also administered the Anxiety, Depression, and Mood Scale (ADAMS), a parent-rated questionnaire designed to assess affective disorders in children with intellectual and developmental disabilities. The questionnaire includes 28 items grouped into five domains: manic/hyperactive behavior, depressed mood, social avoidance, general anxiety, and obsessive behavior [20]. In this study, scores in individual domains were utilized for analysis.

### Cognitive assessments

Several participants with Rett syndrome (*n* = 49) were administered the Mullen Scales of Early Learning [21]. Scores were used to predict age-equivalents in months, which were then divided into the girl’s actual age in months to determine an “age quotient.” Age quotients were then used to assess development across four domains: receptive language, expressive language, visual reception, and fine motor skills.

### EEG data acquisition

Continuous EEG data were acquired for 5 to 10 min in a dimly lit, electrically shielded room. A research assistant ensured that the child remained calm during recording by showing a movie of her choice. Participants were not otherwise engaged during recording. Data were collected using a 128-channel Hydrocel Geodesic Sensor Net System (Electrical Geodesics, Inc., Eugene, OR, USA) and a Net Amps 300 amplifier (EGI, Eugene, OR) via Net Station software (EGI, Eugene, OR). Electrodes positioned on the face (125, 126, 127, and 128) were removed to enhance tolerance of the net. Prior to acquisition, impedances were checked to be below 100 kΩ (within recommended guidelines given high-input impedance capabilities of the amplifier). The data were sampled at either 500 or 1000 Hz, filtered, amplified, and referenced to the vertex (electrode Cz).

### EEG processing

Pre-processing of the data occurred in Net Station (EGI, Eugene, OR). A 1 Hz high pass and 60 Hz notch filter were applied, and any channel with excessive artifact and channels that did not contain EEG data on visual inspection were marked for interpolation. Participants with greater than 10% of total channels marked for interpolation were excluded from further analysis. Data were re-referenced to the average reference after excluding bad channels and exported to MATLAB (r2015a). In MATLAB, data collected at 1000 Hz were downsampled to 500 Hz. The data were detrended using a Kalman filter, and segments containing high-amplitude artifact (> 150 μV) in any channel were excluded from further analysis [22]. Remaining data were broken into 1-s, non-overlapping segments. For analyses involving correlations with the delta band in specifically, EEG data were broken into 2-s, non-overlapping segments and re-run to account for the lower frequencies contained within this band.

### Power spectral analysis

A fast Fourier transform was used to calculate a power spectrum on each segment, and the average power across all 1-s segments for a particular participant was calculated in MATLAB using the Batch Electroencephalography Automated Processing Platform (BEAPP) described by Levin et al. [23]. Tapering with a Hanning window was utilized to attenuate edge artifacts. Results were then binned into frequency bands (delta [2–4 Hz], theta [4–6 Hz], low alpha [6–9 Hz], high alpha [9–13 Hz], beta [13–30 Hz], and gamma [30–50 Hz]) [14, 23]. Power spectra were visualized utilizing the methods and MATLAB code previously described by Cornelissen et al. [24]

**Fig. 1:**
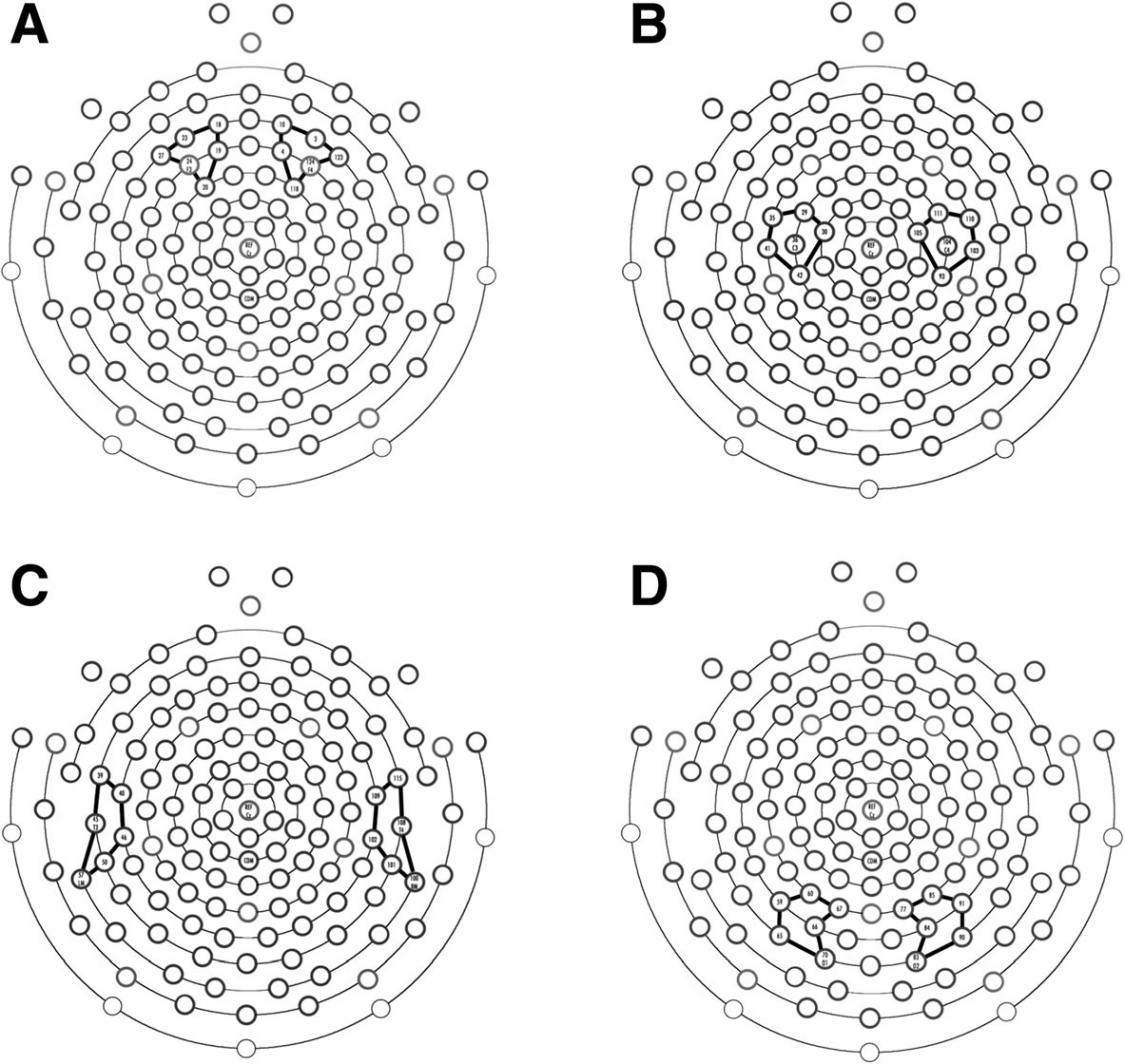
Regions of interest utilized for power spectral analysis. Brain regions and electrode groupings were established based on prior literature and through mathematically determining the five closest electrodes to the electrodes associated with the underlying brain region based on the International 10–20 system. **a** Frontal electrodes included 18, 19, 20, 23, 24 (F3), and 27 on the left and 3, 4, 10, 118, 123, and 124 (F4) on the right. **b** Central electrodes included 29, 30, 35, 36 (C3), 41, and 42 on the left and 93, 103, 104 (C4), 105, 110, and 111 on the right. **c** Temporal electrodes included 39, 40, 45 (T3), 46, 50, and 57 on the left and electrodes 100, 101, 102, 108 (T4), 109, and 115 on the right. **d** Occipital electrodes included 59, 60, 65, 66, 67, and 70 (O1) on the left and 77, 83 (O2), 84, 85, 90, and 91 on the right

For further analysis of power in different regions of the brain, several regions of interest (ROIs) were established (Fig. 1). A frontal ROI was chosen based on those previously reported by Tierney et al. [14] To investigate the regional specificity of EEG characteristics in girls with Rett syndrome, other ROIs in the occipital, central, and temporal region were also established by calculating the closest electrodes to O1 and O2, C3 and C4, and T3 and T4, respectively [25].

In order to determine the effect of 1/ƒ noise on the power spectrum in girls with Rett syndrome, the slope of the power spectrum was estimated via linear regression in log-log space. To control for the effect of high-frequency noise sources in the EEG, particularly movement of the scalp and eye muscles, and because the effect of significant enough changes in 1/ƒ noise can affect the power spectrum even at lower frequencies, the slope of the power spectrum was estimated in the 2 to 24 Hz frequency range. These methods were previously described by Voytek et al. [26]

### Statistical analysis

For the comparison of spectral power values between groups, raw values were log transformed in order to control for the 1/ƒ distribution characteristic of human EEG recordings [14, 25, 27]. Average power in each frequency band in each ROI was then determined for each subject. For the analysis of EEG stability over time, change scores were calculated by subtracting power at visit 2 from power at visit 1. Change scores were then compared between groups.

Statistical analysis was carried out using SPSS Statistics v24.0 software (IBM, Armonk, NY, USA). Nonparametric analyses were utilized due to non-normal distribution of EEG data (based on Shapiro-Wilk tests, *p* < .05 and visual analysis of histograms generated from the data) and/or sample size. Mann-Whitney *U* tests were used to compare PSD and slope values between TD controls and girls with RTT. Kruskal-Wallis tests were used to compare PSD values and slope among TD controls and girls with RTT grouped by regression status (AR and PR), and post-hoc analyses were carried out with Mann-Whitney *U* tests. Mann-Whitney *U* tests were utilized to compare change scores of girls with RTT and TD controls and to compare power values in each frequency band for TD controls and girls with RTT and both the first and second time points. Spearman’s rho correlations were performed to investigate the associations between power values in girls with RTT and age, clinical severity score, scores on the ADAMS subscales, and age quotients on each of the subscales of the Mullen Scales of Early Learning (MSEL). The significance threshold was set to *p* = 0.05 unless otherwise noted.

## Results

### Clinical, behavioral, and cognitive assessments

#### Mullen Scales of Early Learning

Medians and interquartile ranges for developmental quotients for each subscale of the MSEL are reported for all participants with RTT with both MSEL scores and usable EEG data at the first study visit (Table 1). Results are also reported for girls known to be AR or PR at the time of EEG data collection. Mullen developmental quotients were significantly lower in PR girls with RTT in the receptive language (*U* = 85, *z* = − 3.483, *p* < 0.0005), visual reception (*U* = 68.5, *z* = − 4.043, *p* < 0.0005), expressive language (*U* = 56.5, *z* = − 4.321, *p* < 0.0005), and fine motor (*U* = 117.5, *z* = − 2.908, *p* = 0.004) domains.

**Table 1:** Results of cognitive and clinical assessment in girls with RTT by disease stage

	All RTT	AR	PR
Mullen Scales of Early Learning	*n* = 49	*n* = 18	*n* = 27
Visual reception	0.36 (0.46)	0.62 (0.36)	0.24 (0.19)
Receptive language	0.17 (0.13)	0.23 (0.10)	0.11 (0.09)
Expressive language	0.41 (0.55)	0.77 (0.46)	0.26 (0.29)
Fine motor	0.13 (0.18)	0.25 (0.29)	0.09 (0.11)
Clinical severity score	*n* = 47	*n* = 20	*n* = 27
Severity score	20 (8)	21 (7)	19 (7.5)
Anxiety, Depression, and Mood Scale	*n* = 46	*n* = 17	*n* = 26
Manic/hypermanic behavior	6.5 (5)	8 (4)	6.5 (4)
Depressed mood*	3 (4.5)	3 (5.25)	3 (3.5)
Social avoidance	4 (4)	4 (3)	6 (3.75)
General anxiety	5 (3.75)	4 (4)	6 (2.75)
Obsessive behavior	3 (2.75)	3 (2)	4 (2.75)

*RTT n = 39, *PR n = 23*

Data are presented as median (interquartile range). MSEL values are presented as developmental quotients, while CSS and ADAMS data are presented as raw scoresMedian (and interquartile range) developmental quotients based on performance on MSEL domains as well as scores on the ADAMS subscales and clinical severity scores (CSS) are reported for all participants with RTT with usable EEG data at the initial study visit. Values for participants in active regression and that are postregression are also reported. Developmental quotients were calculated by dividing the “developmental age” in a given domain as predicted by performance on the MSEL by the participant’s actual age (in months)

#### Clinical severity score

Medians and interquartile ranges for CSS are reported for all participants with RTT with both clinical assessment data as well as EEG data at the first study visit (Table 1). Results are also reported for girls known to be in active regression and those that were postregression at the time of data collection. No significant differences in CSS were seen between AR and PR girls with RTT. There was no significant correlation between age and CSS.

#### Anxiety, Depression, and Mood Scale

Medians and interquartile ranges for scores on each domain of the Anxiety, Depression, and Mood Scale (ADAMS) are reported for all participants with RTT with both ADAMS scores and usable EEG data at the first study visit (Table 1). No significant differences in ADAMS subscale scores were seen between AR and PR RTT participants. There were no significant correlations between age and score on any ADAMS subscale.

### Power spectral density by clinical diagnosis (RTT versus TD)

**Fig. 2:**
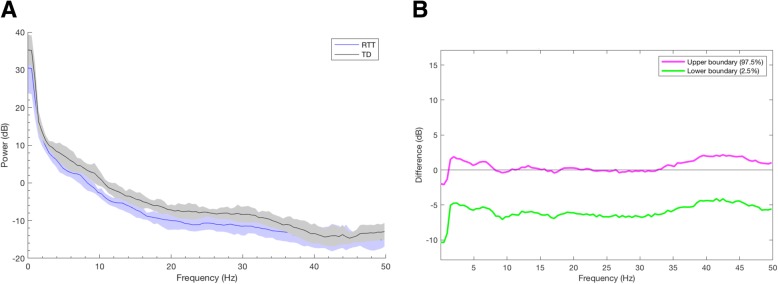
Power spectrum based on clinical diagnosis (RTT or TD). **a** Frontal power spectrum demonstrating that girls with RTT (blue) have decreased power in the lower to middle frequency bands when compared to TD controls (black). **b** Differences in frontal power spectra presented with 95% CI from bootstrap analysis

**Fig. 3:**
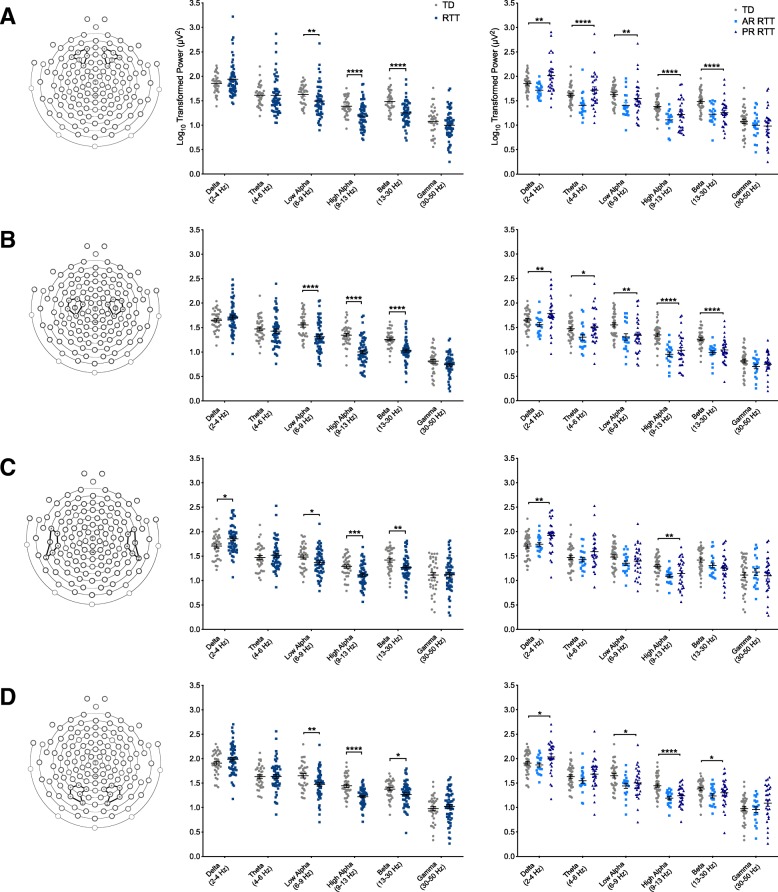
Power spectral density in each ROI by clinical diagnosis. Power (log10 transformed) in each frequency band compared based on clinical diagnosis (RTT versus TD) (left panel) as well as disease stage, with active regression (*n* = 20) defined as experiencing a significant skill loss within 12 months of data collection and postregression (*n* = 29) defined as having no significant skill loss during this time period (right panel). Data are presented as individual power values with lines representing mean with standard error of the mean. To correct for multiple comparisons, a *p* value of 0.002 (noted by three asterisks) was used to determine significance when comparing groups by clinical diagnosis and a *p* value of 0.0005 (noted by four asterisks) was used when comparing groups by disease stage. **a** Power in the frontal ROI. **b** Power in the central ROI. **c** Power in the temporal ROI. **d** Power in the occipital ROI. *TD* typically developing, *RTT* Rett syndrome, *AR* active regression, *PR* postregression. Asterisks indicate significance. *p < 0.05; **p < 0.01; ***p < 0.002; ****p < 0.0005

Log-transformed power was calculated for each participant and compared between TD controls (*n* = 37) and girls with RTT (*n* = 57) in each of four predetermined ROIs (Figs. 2 and 3). To correct for multiple comparisons, a *p* value of 0.002 was used; while only corrected significant differences are noted in the text, all significant *p* values are represented in Fig. 2.

#### Frontal ROI

Girls with RTT demonstrated significantly lower power in the high alpha (*U* = 537, z = − 4.005, *p* < 0.0005) and beta (*U* = 502, *z* = − 4.276, *p* < 0.0005) frequency bands. No significant differences were seen in the delta, theta, low alpha, or gamma bands. Girls with Rett syndrome also demonstrated significantly higher relative power in the delta band (*U* = 1503, *z* = 3.680, *p* < 0.0005) and significantly lower relative power in the high alpha (*U* = 557, *z* = − 3.850, *p* < 0.0005) and beta (*U* = 625, *z* = − 3.247, *p* = 0.001) bands.

#### Central ROI

Girls with RTT demonstrated significantly lower power in the low alpha (*U* = 580, *z* = − 3.672, *p* < 0.0005), high alpha (*U* = 352, *z* = − 5.437, *p* < 0.0005), and beta (*U* = 396, *z* = − 5.096, *p* < 0.0005) frequency bands. No significant differences were seen between groups in the other frequency bands. Girls with Rett syndrome also demonstrated significantly higher relative power in the delta band (*U* = 1708, z = 5.058, *p* < 0.0005) and significantly lower relative power in the low alpha (*U* = 569, *z* = − 3.757, *p* < 0.0005) and high alpha (*U* = 301, *z* = − 5.831, *p* < 0.0005) bands.

#### Temporal ROI

Girls with RTT demonstrated significantly lower power in the high alpha band (*U* = 627, *z* = − 3.308, *p* = 0.001). No significant differences were seen between groups in the other frequency bands. Girls with Rett syndrome demonstrated significantly higher relative power in the delta band (*U* = 1,576, *z* = 4.036, *p* < 0.0005) and significantly lower relative power in the low alpha (*U* = 563, *z* = − 3.804, *p* < 0.0005), high alpha (*U* = 311, *z* = − 5.754, *p* < 0.0005), and beta (*U* = 590, *z* = − 3.595, *p* < 0.0005) bands.

#### Occipital ROI

Girls with RTT demonstrated significantly lower power in the high alpha band (*U* = 463, *z* = − 4.578, *p* < 0.0005). No significant differences were seen between groups in the other frequency bands. Girls with RTT also demonstrated significantly higher relative power in the delta band (*U* = 1,498, *z* = 3.423, *p* = 0.001) as well as significantly lower relative power in the low alpha (*U* = 447, *z* = − 4.702, *p* < 0.0005) and high alpha (*U* = 353, *z* = − 5.429, *p* < 0.0005) bands.

### Power spectral density by disease stage (AR RTT, PR RTT, TD)

We conducted a Kruskal-Wallis test to investigate differences in EEG power between TD controls (*n* = 37) and girls with RTT in active regression (AR, *n* = 20) or that were postregression (PR, *n* = 29) (Fig. 3). To correct for multiple comparisons, a *p* value of 0.0005 was used; while only corrected significant differences are noted in the text, all significant *p* values are represented in Fig. 2.

#### Frontal ROI

There was a significant effect of group on the frontal theta (*H* [2] = 15.204, *p* < 0.0005), high alpha (*H* [2] = 19.769, *p* < 0.0005), and beta (*H* [2] = 18.843, *p* < 0.0005) frequency bands. Post-hoc analyses demonstrated a significant different between the TD and AR groups in the theta (*U* = 159, *z* = 03.528, *p* < 0.0005), high alpha (*U* = 96, *z* = − 4.582, *p* < 0.0005), and beta (*U* = 142, *z* = − 3.812, *p* < 0.0005) bands. When relative power was compared, there was a significant effect of group on the low alpha (*H* [2] = 17.918, *p* < 0.0005) and high alpha (*H* [2] = 17.5, *p* < 0.0005) bands, with girls in the PR group demonstrating significantly lower relative power in these bands.

#### Central ROI

There was a significant effect of group on the central high alpha (*H* [2] = 29.688, *p* < 0.0005) and beta (*H* [2] = 27.494, *p* < 0.0005) frequency bands. Post-hoc analyses demonstrated a significant different between the TD and AR groups in the high alpha (*U* = 59, *z* = − 5.200, *p* < 0.0005) and beta (*U* = 82, *z* = − 4.816, *p* < 0.0005) bands. A significant difference was also seen between TD controls and PR RTT participants in the high alpha (*U* = 235, *z* = − 3.895, *p* < 0.0005) and beta (*U* = 232, *z* = − 3.934, *p* < 0.0005) bands. When relative power was compared, there was a significant effect of group on the delta (*H* [2] = 25.342, *p* < 0.0005), low alpha (*H* [2] = 16.555, *p* = 0.0005), and high alpha (*H* [2] = 33.509, *p* < 0.0005) bands, with girls in the PR group demonstrating significantly higher power in the delta band and lower relative power in the low and high alpha bands.

#### Temporal ROI

No significant group differences were seen in absolute power in any frequency band. There were no significant differences when relative power was compared between groups.

#### Occipital ROI

There was a significant effect of group on the occipital high alpha band (*H* [2] = 21.557, *p* < 0.0005). Post-hoc analyses demonstrated a significant different between the TD and AR groups in the high alpha band (*U* = 160, *z* = − 3.511, *p* < 0.0005). When relative power was compared, there was no significant effect of group on relative power in any frequency band.

### EEG stability across visits

**Fig. 4:**
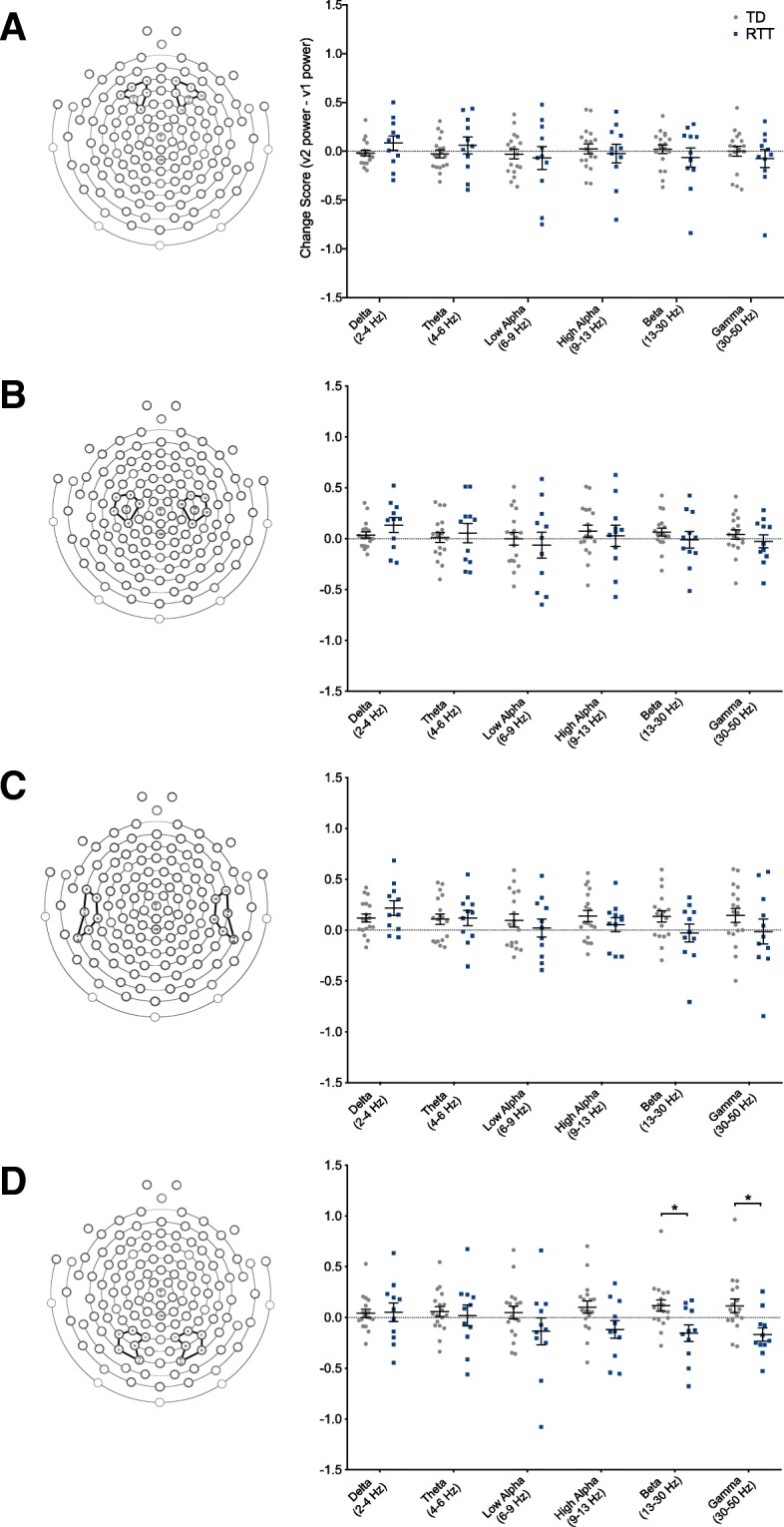
EEG stability over time in girls with RTT versus TD controls. Change scores were calculated by subtracting power (log10 transformed) in each frequency band at visit 1 from power at visit 2. Data are reported as individual values with lines representing the mean and standard error of the mean. **a** Change scores in girls with RTT vs. TD controls in the frontal ROI. **b** Change scores in girls with RTT vs. TD controls in the central ROI. **c** Change scores in girls with RTT vs. TD controls in the temporal ROI. **d** Change scores in girls with RTT vs. TD controls in the occipital ROI. *TD* typically developing, *RTT* Rett syndrome. Asterisks indicate significance. *p < 0.05

Given our interest in assessing changes in EEG power over time, we examined the stability of EEG spectra from the first to second study visit in a subset of participants. Change scores were calculated and compared between groups utilizing independent-samples Mann-Whitney *U* tests (Fig. 4).

#### Frontal, central, and temporal ROIs

No difference in change scores was found between groups in any frequency band.

#### Occipital ROI

Girls with RTT had significantly lower change scores than TD controls in the beta (*U* = 47, *z* = − 2.337, *p* = 0.019) and gamma (*U* = 38, *z* = 2.742, *p* = 0.006) bands, though these changes are not significant if corrected for multiple comparisons (*p* < 0.002).

### Developmental trajectories of EEG power

**Fig. 5:**
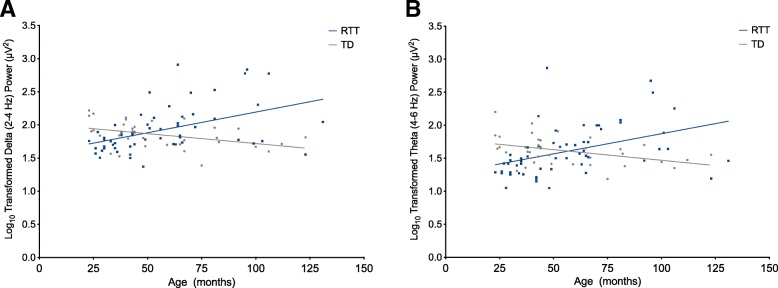
Increased low-frequency frontal power is seen with increasing age in girls with Rett syndrome. Spearman’s rho correlations between age and frontal low frequency power in girls with Rett syndrome and TD controls. Girls with Rett syndrome demonstrate opposite trajectories of baseline EEG frontal power with age in the delta (2–4 Hz) band (**a**) and theta (4–6 Hz) band (**b**) when compared to typically developing controls, with power increasing with age in girls with RTT and decreasing with age in controls. Linear regression was used to generate fit lines for each data set. *TD* typically developing; *RTT* Rett syndrome

To further examine how frontal EEG power changes with age in girls with RTT, we utilized Spearman correlations to relate age to frontal power in all frequency bands (Fig. 5). In girls with RTT, increased age correlated with increased delta power (*r*_s_ = 0.529, *N* = 57, *p* < 0.0005) and theta power (*r*_s_ = 0.552, *N* = 57, *p* < 0.0005). The positive correlation of age with delta power is consistent when power spectral analysis with two-segment EEG segments is utilized (*r*_s_ = 0.424, *N* = 57, *p* < 0.0005). In TD controls, increased age is correlated with decreased frontal delta (*r*_s_ = − 0.435, *N* = 37, *p* = 0.007) and theta (*r*_s_ = − 0.393, *N* = 37, *p* = 0.016) power, though the trend of decreasing delta power is not statistically significant when two-second EEG segments are used in the power spectral analysis (*r*_s_ = − 0.169, *N* = 37, *p* = 0.159).

### Correlations between frontal EEG power and performance on cognitive and clinical assessments

To investigate the association between EEG power and clinical, behavioral, and cognitive measures in girls with RTT, Spearman’s rho correlations were used to relate frontal EEG power and clinical severity scores, scores on subscales of the ADAMS, and performance on the MSEL.

#### Clinical severity score

No correlations were seen between clinical severity score (CSS) and frontal power in any frequency band.

#### Anxiety, Depression, and Mood Scale

No correlations were seen between frontal power and scores on any of the Anxiety, Depression, and Mood Scale (ADAMS) subscales.

#### Mullen Scales of Early Learning

**Fig. 6:**
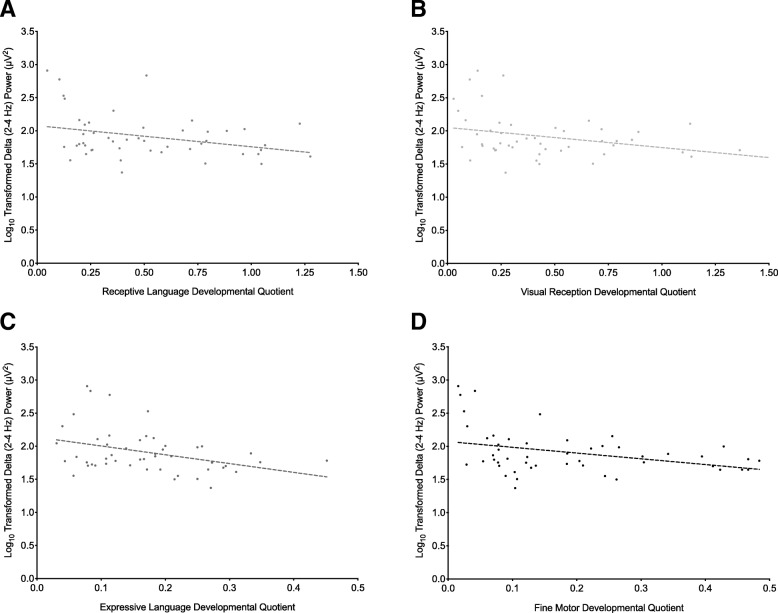
Increased delta power correlates with decreased developmental quotient on the Mullen Scales of Early Learning in girls with Rett syndrome. Spearman’s rho correlations (with fitted line) between frontal delta power and developmental quotient on the Mullen Scales of Early Learning (MSEL) in the receptive language (**a**), visual reception (**b**), expressive language (**c**), and fine motor (**d**) domains. Developmental quotients were calculated by dividing developmental age as predicted by MSEL scores in each domain by chronological age (in months)

Higher frontal delta power was correlated with lower developmental quotients in the visual reception (*r*_s_ = − 0.328, *N* = 49, *p* = 0.021), receptive language (*r*_s_ = − 0.293, *N* = 49, *p* = 0.043), expressive language (*r*_s_ = − 0.354, *N* = 49, *p* = 0.013), and fine motor (*r*_s_ = − 0.348, *N* = 49, *p* = 0.014) domains (Fig. 6). When 2-s EEG segments were utilized in power spectral analysis, higher frontal delta was consistently correlated with lower developmental quotients in the visual reception (*r*_s_ = − 0.315, *N* = 49, *p* = 0.014), receptive language (*r*_s_ = − 0.468, *N* = 48, *p* < 0.0005). Increased theta power correlated with lower developmental quotients in the visual reception (*r*_s_ = − 0.299, *N* = 49, *p* = 0.037), expressive language (*r*_s_ = − 0.366, *N* = 49, *p* = 0.010), and fine motor (*r*_s_ = − 0.300, *N* = 49, *p* = 0.036) domains. No association was found between Mullen Scales of Early Learning (MSEL) developmental quotients and power in the low alpha, high alpha, beta, or gamma bands.

### 1/ƒ slope calculations

**Fig. 7:**
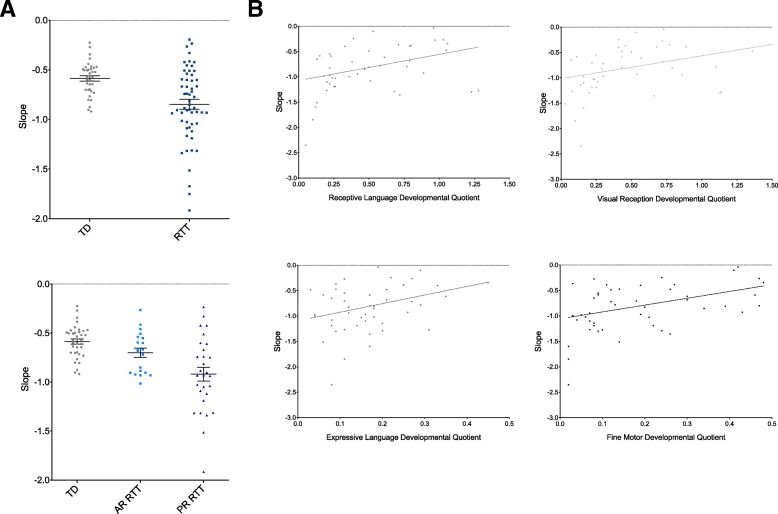
Girls with Rett syndrome demonstrate a significantly more negative 1/ƒ slope of the power spectrum when compared to typically developing controls. **a** The slope of the power spectrum was calculated and compared between girls with RTT and TD controls (top panel) as well as among TD controls, girls with RTT in active regression (AR), and girls with RTT that are postregression (PR) (bottom panel). **b** For girls with RTT, Spearman’s rho correlations (with fitted line) were utilized to relate slope to performance on the Mullen Scales of Early Learning (MSEL) in the receptive language, visual reception, fine motor, and expressive language domains (clockwise from top left). *TD* typically developing, *RTT* Rett syndrome, *AR* active regression, *PR* postregression

To investigate the potential role of abnormal excitatory/inhibitory balance in girls with Rett syndrome and the effect of neural noise in shaping the power spectrum seen in this population, the 1/ƒ slope of the power spectrum was calculated and compared among TD girls as well as both AR and PR girls with RTT (Fig. 7).

Girls with RTT demonstrated a significantly more negative slope when compared to TD controls in both the frontal ROI (*U* = 540, *z* = − 3.982, *p* < 0.000) as well as across the entire brain (*U* = 568, *z* = − 3.765, *p* < 0.000). When girls with RTT were separated based on regression status, girls with RTT that were postregression at the time of EEG recording demonstrated a significantly more negative slope than girls with RTT that are actively regressing or TD controls in the frontal ROI (*H* [2] = 22.180, *p* < 0.000) and across the entire brain (*H* [2] = 18.197, *p* < 0.000).

In girls with RTT, a more positive slope was correlated with improved performance on the visual reception (*r*_s_ = 0.322, *N* = 49, *p* = 0.012), receptive language (*r*_s_ = 0.347, *N* = 48, *p* = 0.008), expressive language (*r*_s_ = 0.315, *N* = 49, *p* = 0.014), and fine motor (*r*_s_ = 0.381, *N* = 49, *p* = 0.003) domains of the MSEL.

## Discussion

Due to the nature and severity of the symptoms seen in girls with Rett syndrome, many current assessments of disease severity and cognition in girls with the disorder are subjective and based largely on provider or parent observation. As a result, there remains a need for a reliable, noninvasive, objective biomarker for use in the assessment of individuals with Rett syndrome. Here, we attempt to characterize the baseline (resting) power spectrum in girls with Rett syndrome not only to better understand the disease and its effects on the brain but also as a first step in identifying unique characteristics that could potentially be utilized in the future assessment and treatment of girls with Rett syndrome.

Various features of the EEG have previously been explored in girls with Rett syndrome. Visual analysis of the background EEG in girls with Rett syndrome has found rhythmic “slowing” as well as gross morphological characteristics such as spike-wave complexes and epileptiform activity [28–30]. Additionally, previous studies of event-related potentials in girls with RTT and mouse models of the disorder have demonstrated abnormalities in both visual evoked potentials (VEPs) and auditory evoked potentials (AEPs) when compared to typically developing children [31, 32]. Our study expands upon this previous work and provides a more in-depth quantitative analysis of spectral power characteristics of girls with Rett syndrome in order to systematically quantify and characterize the differences seen in the baseline EEG of girls with RTT and their associations with various clinical measures currently used in the assessment of individuals with this disorder.

Though girls with RTT did not have significantly higher power in the low-frequency delta and theta bands as compared to TD controls, they did have significantly lower power in the middle-frequency bands as well as significantly higher relative power in the delta band, consistent with a greater contribution of low-frequency activity to the overall EEG previously described in girls with RTT [29, 30]. Girls with RTT also demonstrated a significantly more negative slope of the power spectrum in the lower to middle frequency range, with a more pronounced effect seen in girls that are postregression. These findings mathematically confirm what is seen upon visual inspection of the background EEG in individuals with Rett syndrome. The EEG in Rett syndrome has previously been shown to deteriorate as an individual progresses through the various stages of the disease, with features such as slowing, epileptiform activity, and continuous, slow, spike-wave activity developing as the disease progresses [29]. Similar changes, including increased slow-wave activity and abnormal discharges, can be seen in encephalopathies regardless of the underlying etiology [33]. Differences in power in multiple brain regions have been associated with altered sensory processing, motor function, and communication between different regions of the brain [34]. Thus, these findings could be associated with some of the cognitive, motor, and sensory differences seen in girls with RTT.

An increase in relative delta power in girls with Rett syndrome with no differences in absolute delta power when compared to typically developing children suggest a decrease in overall EEG power in girls with this disorder. This is consistent with molecular findings in mouse models of the disorder, which demonstrate not only decreased inhibitory activity but also decreased excitatory activity as well [35]. An increase in delta power is also similar to findings seen in individuals and mouse models of other neurodevelopmental disorders, particularly Angelman’s syndrome [36]. Given the similarity in the underlying molecular mechanisms of these disorders, these findings could suggest a similar mechanism underlying the altered generation of neural oscillations in these disorders [37].

The increase in delta and theta power in girls with RTT with age, when compared to the decrease in power seen in TD girls with increasing age, suggests that girls with RTT and TD controls demonstrate distinctly different trajectories of development of the power spectrum throughout childhood and development. The decreases in power in the low-frequency delta and theta bands seen with increasing age in TD controls are consistent with studies that have demonstrated an increase in the prevalence of high-frequency oscillations throughout development [38, 39]. The increases in power in the delta and theta frequency bands with age in girls with RTT are similar to EEG changes seen in individuals with neurological disorders such as Parkinson’s and Alzheimer’s disease [13, 40]. These findings are also consistent with previous studies of Rett syndrome that have described a nonspecific “deterioration” of the background EEG with age [6, 16, 28, 41]. On an individual level, this trajectory could possibly be used not only as an index of disease severity or progression but also as an indicator of response to pharmacological and behavioral treatments of RTT, especially given the delta band’s correlation with MSEL performance. However, repeated recordings in the same participants throughout childhood would be necessary to determine whether this trend is observed on an individual level over the course of several years.

Study findings also suggest that increased power in the delta and theta bands in girls with RTT also correlates with worse performance on the MSEL, a measure of multiple aspects of cognitive functioning. The significant difference in MSEL quotients based on disease stage suggests that scores on the MSEL subscales represent a marker of cortical function in girls with RTT that changes reliably with disease progression. As RTT is a progressive neurodevelopmental disorder, it is difficult to separate increasing age from increasing clinical severity, and it is unclear whether the EEG changes that occur throughout development in girls with RTT are a result of aging or progression of the disease. However, the MSEL developmental quotient controls for age, providing a more objective picture of cognitive abilities and cortical function in this population. These results indicate that delta and theta power, or the degree of “slowing” of the background EEG, can be reliably related not only to disease progression in girls with RTT but also to the severity of cognitive dysfunction in girls with the disorder, further supporting the potential for the use of individual trajectories of EEG power as a biomarker in this population.

Though the link between underlying brain physiology and both normal and pathological oscillatory activity observed by EEG remains unclear, numerous studies have investigated the normal and abnormal synaptic interactions and neuronal circuits in the generation of the EEG patterns seen in numerous intellectual and developmental disorders, including Rett syndrome. Research on the cellular basis of evoked potential abnormalities in girls with Rett syndrome has demonstrated that a loss of functional MECP2 from forebrain neurons leads to AEP deficits in mouse models of the disorder [42]. Additionally, regulation of excitatory and inhibitory inputs plays a significant role in the regulation of neural networks, and an abnormal excitatory and inhibitory balance in girls with RTT is thought to be responsible for the abnormalities seen in the VEP and other evoked potentials in girls with RTT [31, 43, 44]. As the generation of evoked potentials is dependent on precise control of excitatory and inhibitory inputs as well as oscillatory activity, it is possible that the molecular abnormalities underlying the ERP abnormalities seen in girls with RTT also contribute to the abnormalities seen in the baseline EEG oscillations, and these abnormal oscillations could contribute to the phenotypic changes seen in mouse models of the disorder and individuals with RTT.

It is also possible that an abnormal excitatory/inhibitory balance within the brain can explain the significant differences in the slope of the PSD seen in girls with Rett syndrome. Previous studies have proposed that the power spectrum is made up not only of rhythmic neural oscillations, a result of synchronous firing of large populations of neurons, but also a 1/ƒ noise component caused by asynchronous firing of neurons that can be estimated from the slope of the power spectrum. Under these assumptions, an increase in neural noise relative to synchronous spiking activity could result in a less-negative slope of the power spectrum, while a decrease in noise and increase in synchronous activity would result in more negative slope [26, 45]. Changes in the balance between neural noise and synchronous oscillatory activity, potentially due to abnormal balance between excitation and inhibition within neural networks, is thought to underlie significant changes observed in the slope of the power spectrum. Girls with Rett syndrome demonstrated a significantly more negative slope of the power spectrum, a finding previously also seen in individuals with schizophrenia, that became more pronounced when the girls were separated based on regression status [45]. Additionally, girls with Rett syndrome demonstrated significant variability in slope of the power spectrum. These findings could potentially represent abnormalities in both excitatory and inhibitory connections in the brain, previously seen in mouse models of the disorder, but could also possibly reflect significant differences in neural connectivity as well as the balance between excitation at the molecular level among individuals with this disorder [35].

This study is not without limitations. Our study provides a cross-sectional view of EEG power in a relatively large group of individuals with Rett syndrome, but participants are varied in terms of age, disease stage, and disease severity. Though differences in EEG power were seen at the group level, the associations between abnormal EEG findings, disease phenotype, and the underlying pathophysiology in the brain are still not well understood. To fully utilize EEG spectral power as a biomarker, further studies utilizing serial EEG recordings as well as measurements of clinical severity and cognitive function in girls with Rett syndrome must be done to determine if the trends reported here can be observed on an individual level over time.

## Conclusions

This study not only demonstrates the feasibility of collecting EEG data in this population but also shows that girls with Rett syndrome have distinct patterns of EEG spectral power when compared to typically developing children. These findings are similar to prior studies that demonstrated a slowing of the EEG in girls with RTT, and our findings indicate that the degree of slowing of the EEG correlates negatively with performance on a measure of cognition in this population. Though more studies are needed to assess the reproducibility of these findings, this increase in delta power, as well as the abnormal spectral slope seen in girls with Rett syndrome, could represent abnormal neural circuitry in Rett syndrome and could potentially be used as a marker of severity in individuals with this disorder due to associations with more severe disease phenotypes and its worsening with disease progression.

## Abbreviations

ADAMS: Anxiety, Depression, and Mood Scale
AEP: Auditory evoked potential
AR: Active regression
CSS: Clinical severity score
CT: Computed tomography
EEG: Electroencephalography
MECP2: Methyl-CpG binding protein 2
MRI: Magnetic resonance imaging
MSEL: Mullen Scales of Early Learning
PR: Postregression
ROI: Region of interest
RTT: Rett syndrome
TD: Typically developing
VEP: Visual evoked potential
